# A new methodical approach in neuroscience: assessing inter-personal brain coupling using functional near-infrared imaging (fNIRI) hyperscanning

**DOI:** 10.3389/fnhum.2013.00813

**Published:** 2013-11-27

**Authors:** Felix Scholkmann, Lisa Holper, Ursula Wolf, Martin Wolf

**Affiliations:** ^1^Biomedical Optics Research Laboratory, Division of Neonatology, University Hospital ZurichZurich, Switzerland; ^2^Institute for Complementary Medicine, University of BernBern, Switzerland

**Keywords:** functional near-infrared imaging, hyperscanning, brain-to-brain coupling, inter-personal brain activity, hyperconnectivity, social neuroscience

## Abstract

Since the first demonstration of how to simultaneously measure brain activity using functional magnetic resonance imaging (fMRI) on two subjects about 10 years ago, a new paradigm in neuroscience is emerging: measuring brain activity from two or more people simultaneously, termed “hyperscanning”. The hyperscanning approach has the potential to reveal inter-personal brain mechanisms underlying interaction-mediated brain-to-brain coupling. These mechanisms are engaged during real social interactions, and cannot be captured using single-subject recordings. In particular, functional near-infrared imaging (fNIRI) hyperscanning is a promising new method, offering a cost-effective, easy to apply and reliable technology to measure inter-personal interactions in a natural context. In this short review we report on fNIRI hyperscanning studies published so far and summarize opportunities and challenges for future studies.

## Introduction

**Figure 1:**
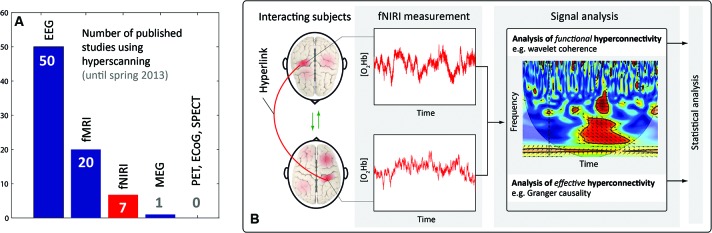
**(A)** Number of published hyperscanning studies in the field of neuroscience (according to an own analysis using PubMed and Google Scholar). EEG, fMRI, fNIRI, magneto-encephalography (MEG), positron emission tomography (PET), electrocorticography (ECoG), single-photon emission computed tomography (SPECT). **(B)** Visualization of important terms in the context of hyperscanning, and illustration of a typical signal processing for analyzing fNIRI hyperscanning data.

A new approach to investigate neuronal correlates of interaction between two or more people is emerging: the measurement of *inter-personal* (between-person) dynamics of brain activity, termed “hyperscanning” (for reviews, see Astolfi et al., 2011; Dumas et al., 2011; Babiloni and Astolfi, 2012; Genvins et al., 2012; Konvalinka and Roepstorff, 2012; Schilbach et al., 2013). This approach constitutes a *third* stage in the development of neuroscience. The *first* stage comprised the classic cognitive neuroscience paradigm, i.e., the measurement of *intra-personal* (within-person) brain activity with a focus on the functional specialization of the individual brain as well as its activity in creating representations of the inner and outer world. The *second* stage emerged from the field of *social neuroscience* and was developed, popularized and formulized in the 1990s by Cacioppo and Berntson (1992) as a “multi-level analysis of social psychological phenomena”. The main methodological approach in social neuroscience is to investigate intra-personal brain dynamics during *inter-personal interactions*. Research in this field revealed that specific brain structures of the “social brain” are involved in social cognition, e.g., brain areas constituting the “mirror neuron system” (Saxe, 2006; Frith, 2007), the “theory of mind” (Premack and Woodruff, 1978; Frith and Frith, 2001) or the “empathy network” (Bernhardt and Singer, 2012). Despite the impressive insights into the neurobiological aspects of human social interaction that have emerged from these two approaches, the neurobiological processes involved in real interpersonal interactions (i.e., the brain-to-brain mechanisms between persons)—which represent the “dark matter” in social neuroscience—cannot be investigated with these methodologies (Przyrembel et al., 2012). As a consequence, the next step in social neuroscience can be regarded as the assessment of the neuronal correlates of social interaction dynamics, and thus as moving from the observer’s perspective towards a truly interactive approach, i.e., “a shift from a single-brain to a multi-brain frame of reference” (Hasson et al., 2012). The measurement of brain activity from two or more people simultaneously, and the quantification of the *inter-personal brain-to-brain coupling* is a methodological tool of particular importance in this approach, which allows the assessment of the bidirectional information flow between interacting persons. This aspect “has been largely neglected” (Hari and Kujala, 2009) in neuroimaging studies so far. The significance of this new step in social neuroscience, i.e., “two-person neuroscience” (Hari and Kujala, 2009), is evident in the growing number papers published about this topic; see for example the special issue titled “Towards a neuroscience of social interaction” published recently in this journal. Brain-to-brain coupling may serve an integral function in social interaction, as for example in a teacher-student (teaching-learning) interaction, where “interpersonal synchronization may support reciprocal, dynamical feedback between teacher and students, through implicit behavioral contagion” (Watanabe, 2013). The first studies using hyperscanning approaches can be traced back to electroencephalography (EEG) studies from the 1960s and 1970s (Duane and Behrendt, 1965; Hearne, 1977). The first hyperscanning study employing functional magnetic resonance imaging (fMRI) was conducted 11 years ago by Montague et al. (2002) who coined the term *hyperscanning*. Connections between two brains were termed *hyperconnections* and each connection a *hyperlink* (see Figure 1A). The first hyperscanning study applying functional near-infrared imaging (fNIRI) was published very recently in 2011 by Funane et al. (2011). So far this research, using EEG, fMRI and fNIRI, showed that brain-to-brain coupling is a non-local emergent phenomenon, i.e., it cannot be reduced to the local activity of a single brain (Hari and Kujala, 2009; Chatel-Goldman et al., 2013). That the “interaction process as a whole has properties that cannot be reduced to the contributions of the isolated agents” was also recently shown by an evolutionary robotics model simulating social interaction (Froese et al., 2013).

The aim of the present paper is (i) to review the fNIRI hyperscanning studies performed so far, and (ii) to summarize opportunities and challenges for future fNIRI hyperscanning studies.

## Beyond individual brain activity: fNIRI hyperscanning

Until spring 2013, seven research papers were published employing the fNIRI hyperscanning methodology. A comparison with the number of hyperscanning studies by other neuroimaging modalities can be found in Figure 1A. All fNIRI studies applied near-infrared spectroscopy (NIRS) devices with more than 4 channels thus enabling near-infrared imaging (NIRI), i.e., measuring changes in oxy- and deoxyhemoglobin concentration ([O_2_Hb] and [HHb], respectively) at different locations (realized by different source-detector channels) of the heads of two subjects simultaneously. For a review on fNIRI, refer to Ferrari and Quaresima (2012) and Scholkmann et al. (2013b). Table 1 depicts the details of these studies.

**Table 1:** **Listing of fNIRI hyperscanning studies performed so far**.

**Reference**	**Task**	**fNIRI setup and probe positions**	**Signal analysis**	**Results**
Funane et al. (2011)	Cooperation	22 ch, R&L-PFC	Cov	Cov ↑
Cui et al. (2012)	Cooperation, competition	22 ch, R&L-PFC	WC	Coop.: WC ↑ Comp.: WC −
Dommer et al. (2012)	Dual n-back	4 ch, L-PFC	WC, BA	WC ↑
Holper et al. (2012)	Imitation	4 ch, L-PFC	WC, GC	WC ↑, GC ↑
Jiang et al. (2012)	Communication	20 ch, L-FTPC, 3 ch L-DPFC	WC (0.01–0.1 Hz)	Face-to-face communication: WC ↑
Duan et al. (2013)	Competition	22 ch, L-SMC	Cor	Cor ↑
Holper et al. (2013)	Teacher–student interaction	4 ch, L-PFC	BA, Cor	Successful teaching, successful learning: activity ↓, Cor ↑

Abbreviations *channels* *(ch)* *right and left* *(R8L)* *left* *(L)* *prefrontal cortex* *(PFC)* *inferior parietal lobule* *(IPL)* *frontal/temporal/parietal cortices* *(FTPC)* *dorsolateral PFC* *(DPFC)* *sensorymotor cortex* *(SMC)* *covariance* *(Cov)* *wavelet coherence* *(WC)* *Granger causality* *(GC)* *block averaging* *(BA)* *correlation* *(Cor)* .

Funane et al. (2011) employed a 22-channel NIRI device to measure simultaneously in two persons changes in [O_2_Hb] and [HHb] in the medial prefrontal cortex (PFC) while performing a cooperative button-press task. Two participants were instructed to synchronize their respective button presses as best as possible. Twelve subjects were measured and the [O_2_Hb] signals were analyzed. The authors reported an increase in the covariance (Cov) of [O_2_Hb] when the subjects successfully interacted in the cooperative task, i.e., when button-presses were highly synchronized. They also found a significant positive correlation between the task performance and the degree of [O_2_Hb] Cov.

Cui et al. (2012) employed also a 22-channel NIRI device to measure [O_2_Hb] and [HHb] changes in two persons simultaneously during two different tasks: a cooperation task, i.e., simultaneous button-pressing, with the aim to reach a smallest possible time difference between the responses of the two subjects, and a competition task, i.e., simultaneous button-pressing, with the aim to respond faster than the competitor. Twenty-two subjects were measured and [O_2_Hb] was analyzed. The brain-to-brain coupling was quantified by calculating the wavelet coherence (WC)—a measure of the cross-correlation of two time series as a function of frequency and time. The authors found that the coherence (in the frequency band 0.08–0.3 Hz) between the two subjects’ right superior frontal cortices increased during the cooperation, but not during the competition task.

Dommer et al. (2012) performed a fNIRI hyperscanning study with two novel 4-channel wireless NIRI-devices (Muehlemann et al., 2008), allowing an unconstrained setting without disturbing cables. Changes in [O_2_Hb] and [HHb] were recorded on the left PFC during performance on a dual n-back task simultaneously in paired players (eight subjects) as compared to single players (seven subjects). Signal analysis was performed on changes in total hemoglobin concentration (tHb) ([tHb] = [O_2_Hb] + [HHb]). Both, the increase in the block-averaged [tHb] hemodynamic response during the tasks as well as the WC were determined. It was found that (i) the hemodynamic response was larger for the paired compared to the single players, and (ii) that inter-personal brain coherence increased during the joint n-back task as compared to baseline. The coherence increase was found in the frequency bands 0.7–4 Hz (related to the heart rate (HR)) and 0.06–0.2 Hz (related to spontaneous low-frequency oscillations (LFOs)), indicating that the joint performance was associated with a synchronization of HR and LFOs.

Holper et al. (2012) investigated, with the same fNIRI-setup as Dommer et al. (2012), how brain-to-brain coupling is influenced during imitation. A paced finger-tapping task was performed by two subjects, where either one of the subjects (i.e., the imitator) had to adapt his/her tapping dynamics to the other one (i.e., the model) (imitation task) or both subjects tapped with the same pacing mode (control task). Sixteen subjects participated in the study. [tHb] changes from the left PFC were analyzed and the WC as well as the GC (a measure of the directionality of influence, see also section Opportunities and challenges) was computed. The authors found an increased coherence (in the frequency bands 0.25–0.5 Hz and 2.5–1 Hz) and increased GC during the imitation task. In addition, the causality analysis showed that the cerebral hemodynamics of the imitator adapted to the ones of the model.

Jiang et al. (2012) performed a fNIRI hyperscanning study with 20 subjects performing four different communication tasks, i.e., a face-to-face dialogue, a face-to-face monologue, a back-to-back dialogue, and a back-to-back monologue. Changes in [O_2_Hb] were measured using a multi-channel NIRI device. An optode with 22 channels was placed over the left side of the head so that the frontal, temporal and parietal cortices were covered. Another optode, with 3 channels, was placed above the left DPFC. Synchronization between the brains was determined by calculating the WC (in the frequency band 0.01–0.1 Hz). The analysis showed that a coherence increase only occurred during the face-to-face dialogue. The increase was observed over the left inferior frontal cortex.

Duan et al. (2013) implemented a “cross-brain neurofeedback” setup which measured the [O_2_Hb] changes over the left parietal (sensorimotor) brain in two subjects with a 22 channel fNIRI device while they performed a competitive task (“tug-of-war” game), with feedback information displayed on a screen. The subjects were told to actively imagine that they were physically participating in the tug of war. On the screen, a rope with a ribbon in it was displayed. The aim of the “tug-of-war” game was to pull the ribbon to the left end of the rope (for subject A) or the right end (for subject B). The position of the ribbon was controlled by the quotient of [O_2_Hb] changes from subject A and B. The online data analysis showed that the subjects were able to control the ribbon position by their brain activity measured online by fNIRI. In an offline analysis, the authors found a decrease in the correlation of the [O_2_Hb] changes (calculated by the Pearson correlation coefficient) from subject A and B when one subject was winning the game, compared to when victory or defeat was not clear. The most recent study was conducted by Holper et al. (2013), employing the same fNIRI-setup as in Dommer et al. (2012). On 17 pairs of subjects, an inter-personal educational dialog task was performed in which subjects performed as teacher-student pairs. For the statistical analysis, both block-averaged hemodynamic activities of [O_2_Hb] and [HHb] were measured on the left PFC and the correlation between the teachers’ and students’ hemodynamic signals was investigated. The analysis revealed that students who successfully acquired knowledge during the dialog had a decreased [O_2_Hb] during the learning phase compared to the others who did not show a transfer of knowledge. The study further demonstrated that teachers and students showed a positive correlation of cerebral hemodynamic activity when the teaching was successful.

In summary, despite the fact that different experimental paradigms, measurement locations and signal analysis methods have been used, in all of the seven summarized fNIRI hyperscanning studies an inter-personal brain-to-brain coupling was demonstrated. Concerning the measurement position in general, the PFC is of particular interest since it has a role in social interaction and particularly in brain-to-brain coupling (Sänger et al., 2011). Further, whereas in two of the studies the brain activity was measured in the left and right cortices (Funane et al., 2011; Cui et al., 2012), in the other five (Dommer et al., 2012; Holper et al., 2012; Jiang et al., 2012; Duan et al., 2013; Holper et al., 2013) it was measured only in regions in the left part of the brain. One the one hand, the restriction of only measuring regions positioned on the left seems to be justified since it is known, for example, that the centers for perceiving and interpreting social information have been associated with increased activity in the left inferior frontal cortex (Pobric and Hamilton, 2006; Keuken et al., 2011). On the other hand, “visual and motor components of the human mirror system are not left-lateralized” (Aziz-Zadeh et al., 2006) and the right temporal parietal junction is involved in “complex social and moral reasoning” (Miller et al., 2010), highlighting the need to measure both cortices in fNIRI hyperscanning experiments. The observed change in coherence in the LFO range observed by several studies can be either explained by a coupling of the autonomic nervous systems since the LFO amplitude changes reflect primary the vasomotor tone of arterial blood vessels modulated by the sympathetic nervous system (Julien, 2006), or by a local modulation of the neuro-vascular coupling due to neural activity.

## Opportunities and challenges

fNIRI hyperscanning bears a great potential for future neuroscience studies since—compared to many other neuroimaging modalities—it offers a cost-effective, easy to apply and reliable technology to measure inter-personal interactions in a more natural context.

One important issue in hyperscanning studies concerns the type of signal processing methods to assess the brain-to-brain coupling. From a neurophysiological point of view, one should distinguish between two types of coupling: *functional* and *effective* hyperconnectivity—in analogy to the functional and effective connectivity typically assessed within one brain (Friston, 1994). Functional (hyper-) connectivity refers to a statistical dependence between variables and can be quantified for example by determining the Cor, the correlation (which is a normalized Cov) or the phase-locking of coherence. Effective (hyper-) connectivity refers to a directed causal interaction which can be determined for example using GC or transfer entropy. From a technical point of view, the signal processing methods for analysis of functional and effective hyperconnectivity can be classified according to methods performed in the (i) time, (ii) frequency, or (iii) time-frequency domain. The WC methodology applied in four of the fNIRI studies (Cui et al., 2012; Dommer et al., 2012; Holper et al., 2012; Jiang et al., 2012) is part of the last mentioned class (iii). Figure 1B sketches a typical fNIRI hyperscanning signal processing in the time-frequency domain. The various signal processing methods developed so far for correlation and causality analysis should be exploited in future studies.

Another important issue concerns the experimental paradigms for hyperscanning studies. As summarized by Babiloni and Astolfi (2012), the paradigms employed so far comprise simple motor tasks (e.g., button pressing), music production, interacting by gesticulation, facial expressions, eye contact, verbal dialogue, synchronizing hand or finger movements, or letting the subjects interact in a game theory context. Creating further paradigms that allow optimum capturing of brain-to-brain coupling is an important task for future studies. One difficulty of tasks that involve rhythmic actions (e.g., button pressing) is that they also elicit rhythmic brain activity, which could be misinterpreted as brain-to-brain coupling. In order to distinguish between a synchronous brain activity due to the task and a brain-to-brain coupling due to real social interaction, it would be of particular importance to assess the *effective* hyperconnetivity, and—most importantly—to use appropriate experimental paradigms with appropriate control conditions. The role of brain-to-brain coupling as a function supporting social interaction beyond the coupling in sensorimotor signals between two people remains to be seen.

In addition, a promising option for future hyperscanning studies is applying different modalities simultaneously, e.g., the combined application of fNIRI with fMRI or EEG, or the combination of fNIRI with the measurement of systemic parameters, e.g., HR, electrodermal activity or changes in respiration. Also the continuous measurement of the blood pressure and arterial CO_2_ may be important to exclude confounding factors in fNIRI measurements, as highlighted recently by Tachtsidis et al. (2009) and Scholkmann et al. (2013a), respectively. The measurement of systemic parameters during hyperscanning studies is not only important in order to exclude confounders but also to elucidate the mechanisms enabling the brain-to-brain coupling. What is known so far is that the interaction between persons includes neuronal and systemic physiological processes, leading to a coupling of not only brain states but also states of the whole physiology, mainly happening unconsciously. Examples for this are the increase in breathing of a person that observes exertion (e.g., weight lifting) (Paccalin and Jeannerod, 2000), postural responses when observing human imbalance (Tia et al., 2011), posture and body movement synchronization (Bernieri, 1988; Chartrand and Bargh, 1999; Sharpley et al., 2001; Yun et al., 2012), and synchronization of HR and respiration (Florian et al., 1998; Mcfarland, 2001; Konvalinka et al., 2011; Xygalatas et al., 2011)—phenomena that are known as *mimicry*, *automatic imitation* (e.g., postural responses due to observation) and *entrainment* (e.g., posture/body and HR/respiration synchronization; Knoblich and Sebanz, 2008; Chartrand and Van Baaren, 2009; Heynes, 2011; Kinsbourne and Helt, 2011).

To improve the sensitivity of the fNIRI measurement to cerebral hemodynamics and oxygenation it would be desirable for future studies to apply methods that reduce the influence of superficial changes on the measured signal. Such methods comprise hardware (e.g., Hueber et al., 1999; Suzuki et al., 1999) or signal processing approaches (e.g., Saager and Berger, 2005). Also the analysis of changes in [HHb], [O_2_Hb] and [tHb], and not only in one signal alone (i.e., [O_2_Hb] or [tHb]), will help to distinguish between systematically and neuronally driven changes.

An inherent limitation of fNIRI is that only cortical brain regions can be accessed. The method is not able to measure sub-cortical areas. However, the fact that important brain regions for social interaction are located in the cerebral cortex makes this limitation less significant.

## Conclusion

fNIRI hyperscanning is a promising new field in social neuroscience with a great potential to gain further insights into the neurobiological correlates of inter-personal interactions. fNIRI studies performed so far using this methodological approach are promising and demonstrated the feasibility of fNIRI for hyperscanning. We suggest for future studies (i) to exploit the variety of signal processing methods already available for quantifying the between-brain coupling and improving the signal quality, and (ii) to realize multi-modal fNIRI hyperscanning measurements by combining fNIRI with other neuroimaging or physiological measurements.

## Conflict of interest statement

The authors declare that the research was conducted in the absence of any commercial or financial relationships that could be construed as a potential conflict of interest.
